# The complete chloroplast genome sequence of *Onosma paniculatum* Bur. et Franch. (Boraginaceae), a medicinal plant in Yunnan and its adjacent regions

**DOI:** 10.1080/23802359.2019.1673230

**Published:** 2019-10-04

**Authors:** Qi Chen, Dequan Zhang

**Affiliations:** a College of Pharmacy and Chemistry, Dali University, Dali, Yunnan, China;; b Institute of Materia Medica, Dali University, Dali, Yunnan, China;; c Key Laboratory of Yunnan Provincial Higher Education Institutions for Development of Yunnan Daodi Medicinal Materials Resources, Yunnan, China

**Keywords:** *Onosma paniculatum*, Boraginaceae, complete chloroplast genome, Illumina sequencing, phylogenetic analysis

## Abstract

*Onosma paniculatum* is a medicinal plant commonly used in Yunnan and its adjacent regions, China. In the present study, we sequenced the complete chloroplast (cp) genome sequence of *O. paniculatum* to investigate the phylogenetic relationship in the Tubiflorae. The total length of the chloroplast genome was 151,198 bp, with 37.4% overall GC content and exhibited typical quadripartite structure, a pair of IRs (inverted repeats) of 25,889 bp was separated by a small single copy (SSC) region of 17,274 bp and a large single copy (LSC) region of 82,146 bp. The cp genome was composed of 113 genes, including 79 protein coding genes, 30 tRNA genes, and 4 rRNA genes. The phylogenetic analysis indicated that Boraginaceae was closely related to Convolvulaceae and Solanaceae in Tubiflorae.

*Onosma* L. is an important genus of the Boraginaceae family, which includes about 150 species all over the world (Naz et al. 2006). The genus is distributed across Europe to East Asia, especially in Western and Central Asia, the Mediterranean area, Anatolia and Southeast Europe (Kolarčik et al. 2010). In this genus, there are 29 species in China (Zhu et al. 1995), and some species of them, such as *Onosma paniculatum* Bur. et Franch., possess important medicinal values and are commonly used as the alternative of Arnebiae radix in Yunnan and its adjacent regions, China (Ge et al. 2003; Chinese Pharmacopoeia Commission 2015). Until now, most of the studies on *Onosma* and its related genus have focused on its chemical compositions, morphological taxonomy and molecular phylogeny (Kumar et al. 2013; Binzet et al. 2018; Nasrollahi et al. 2019); whereas, no complete chloroplast genome sequence has been reported in NCBI and other databases. Therefore, we sequenced the complete chloroplast genome sequence of the important medicinal plant, *O. paniculatum*, as well as reconstructed its phylogenetic tree with other groups based on cp genomes.

Molecular materials and voucher specimen (No. ZDQ105) of *O. paniculatum* were collected from Dali county, Yunnan, China (N25.76°, E100.23°), and then deposited at the Herbarium of Medicinal Plants and Crude Drugs of the College of Pharmacy and Chemistry, Dali University (DLUSYX19001). The total genomic DNA was extracted using the improved CTAB method (Doyle 1987; Yang et al. 2014), and sequenced with Illumina Hiseq 2500 (Novogene, Tianjing, China) platform with pair-end (2 × 300 bp) library. About 6.34 Gb of raw reads with 21,147,864 paired-end reads were obtained from high-throughput sequencing. The raw data was filtered using Trimmomatic v.0.32 with default settings (Bolger et al. 2014). Then paired-end reads of clean data were assembled into circular contigs using GetOrganelle.py (Jin et al. 2018). Finally, the cpDNA was annotated by the Dual Organellar Genome Annotator (DOGMA; http://dogma.ccbb.utexas.edu/) (Wyman et al. 2004) and tRNAscan-SE (Lowe and Chan 2016).

The annotated chloroplast genome was submitted to the GenBank with accession number MN175501. Total length of the chloroplast genome was 151,198 bp, with 37.4% overall GC content. With typical quadripartite structure, a pair of IRs (inverted repeats) of 25,889 bp was separated by a small single copy (SSC) region of 17,274 bp and a large single copy (LSC) region of 82,146 bp. The cp genome was composed of 113 genes, including 79 protein coding genes, 30 tRNA genes, and 4 rRNA genes. Among them, 17 genes were duplicated in the inverted repeat regions, 16 genes, and 6 tRNA genes contain one intron, while 2 genes (*ycf3* and *clpP*) have two introns.

To this day, there is nearly no annotated complete chloroplast genome for species in *Onosma* and its related genus. To investigate its phylogeny, a total of 52 cp genome sequences of species belonged to the Order Tubiflorae, were downloaded from the NCBI database used for phylogenetic analysis. After using MAFFT V.7.149 for aligning (Katoh and Standley 2013), jModelTest v.2.1.7 (Darriba et al. 2012) was used to determine the best-fitting model for the chloroplast genomes. Then Bayesian inference (BI) was performed by MrBayes v.3.2.6 (Ronquist et al. 2012) with *Fritillaria cirrhosa* D. Don (No. KF769143) as outgroup. The results showed that Boraginaceae was closely related to Convolvulaceae and Solanaceae, and the three families formed into a monophyletic clade which was divided out in the early evolution of Tubiflorae (Figure 1). Furthermore, complete chloroplast genome of *O. paniculatum* would be beneficial to phylogeny of Boraginaceae and its related families, as well as developing chloroplast markers for further studies.

**Figure 1.**
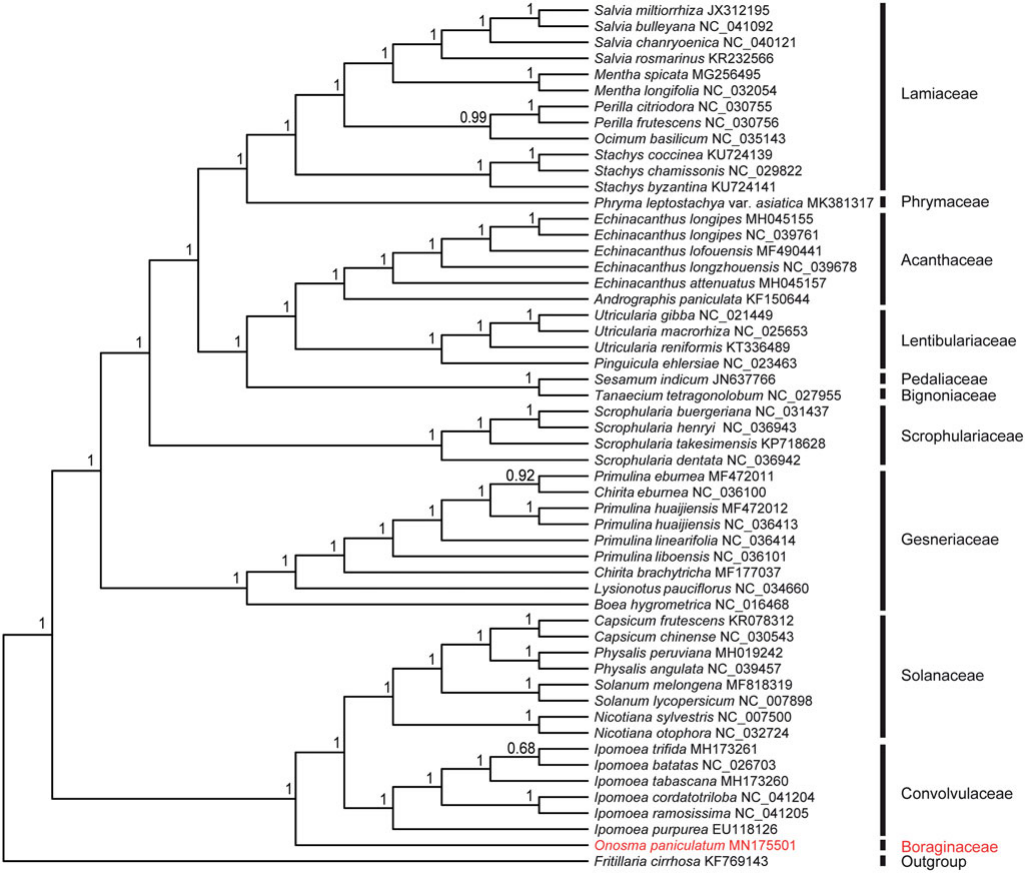
Phylogeny of 53 species of the complete chloroplast genome sequences within the order Tubiflorae based on the Bayesian inference (BI). The GTR + G + I model was employed as the best-fit nucleotide substitution model as suggested using Mr. Bayes 3.2.6 with *Fritillaria cirrhosa* D. Don (No. KF769143) as an outgroup.
